# Effect of calcitriol on serum hepcidin in individuals with chronic kidney disease: a randomized controlled trial

**DOI:** 10.1186/s12882-018-0823-7

**Published:** 2018-02-09

**Authors:** Bhupesh Panwar, Diane McCann, Gordana Olbina, Mark Westerman, Orlando M. Gutiérrez

**Affiliations:** 1 Departments of Medicine, University of Alabama at Birmingham, ZRB 522, 1720 2nd AVE S, Birmingham, AL 35294-0006 USA; 2 Departments of Epidemiology, University of Alabama at Birmingham, Birmingham, AL USA; 3 Intrinsic LifeSciences, La Jolla, CA USA

**Keywords:** Calcitriol, Hepcidin, Chronic kidney disease, Vitamin D, Anemia of CKD

## Abstract

**Background:**

Anemia is highly prevalent in chronic kidney disease (CKD). Elevated hepcidin concentrations are an important mediator of disordered iron metabolism, a key mechanism underlying anemia of CKD. Vitamin D was recently shown to reduce serum hepcidin concentrations in healthy individuals. We examined whether treatment with calcitriol reduces serum hepcidin in individuals with CKD.

**Methods:**

A total of 40 participants with stage 3 or 4 CKD (eGFR 15–60 ml/min/1.73m^2^) were randomized to receive either oral calcitriol 0.5 mcg daily or identically-matched placebo for 6 weeks. The primary outcome variable was change in serum hepcidin concentrations. Secondary outcomes variables included the change in iron parameters, calcium, phosphorus, intact parathyroid hormone and hemoglobin concentrations. Study samples were drawn at baseline, 3 days, 1 week, 4 weeks and 6 weeks after randomization. Repeated measures analysis was used to examine differences in outcome variables over time in the two groups.

**Results:**

There were no significant differences in the baseline characteristics between the placebo and calcitriol arms. Over 6 weeks of follow-up there were no significant differences in the change in serum hepcidin, iron parameters, or hemoglobin between the two groups. Serum calcium and phosphorus significantly increased and PTH significantly decreased after 6 weeks in calcitriol group whereas these analytes did not change in the placebo group.

**Conclusion:**

Calcitriol did not reduce serum hepcidin concentrations among individuals with mild to moderate CKD. Future studies are needed to assess if nutritional forms of vitamin D affect hepcidin concentrations in CKD.

**Trial registration:**

ClinicalTrials.gov Identifier: NCT01988116. Registered: November 4, 2013.

**Electronic supplementary material:**

The online version of this article (10.1186/s12882-018-0823-7) contains supplementary material, which is available to authorized users.

## Background

Anemia is highly prevalent in chronic kidney disease (CKD), affecting nearly 5 million CKD patients in the United States alone (~ 15% of the US CKD population) [1], and representing a substantial economic burden [2]. Iron deficiency is highly prevalent in CKD and is a major mechanism underlying anemia of CKD. Hepcidin is a hormone that regulates iron homeostasis by blocking iron absorption in the gut and iron efflux from macrophage and hepatocyte stores [3–5]. Serum hepcidin concentrations are elevated in CKD and play a key role in the development of anemia in CKD by reducing iron bioavailability for erythopoiesis [6–9]. As such, lowering hepcidin concentrations has emerged as a potential therapy for improving iron-restricted erythropoiesis in CKD patients.

Vitamin D has recently been shown to regulate hepcidin synthesis through direct transcriptional inhibition of hepcidin expression [10]. The clinical relevance of these data is supported by a small pilot study showing that treatment with ergocalciferol decreased hepcidin concentrations in healthy individuals [10]. Whether vitamin D has similar affects on hepcidin concentrations and iron parameters in individuals with CKD is unclear. Accordingly, we randomized 40 individuals with stage 3 or 4 CKD (estimated glomerular filtration rate [eGFR] 15–60 ml/min/1.73m^2^) to once daily oral calcitriol vs placebo (20 per arm) for 6 weeks. We hypothesized that, as compared to treatment with placebo, treatment with an activated vitamin D analog (calcitriol) would lower hepcidin concentrations and improve markers of iron status in individuals with stage 3 or 4 CKD.

## Methods

### Study design (Fig. 1)

We tested our hypothesis in a randomized, placebo-controlled double-blinded study of 40 participants with stage 3 or stage 4 CKD (eGFR 15–60 ml/min/1.73 m^2^ by CKD-EPI) [11]. Participants were recruited from the nephrology outpatient clinics at the University of Alabama at Birmingham (UAB). Participants 19 years or older with stage 3 or stage 4 CKD were eligible for our study. Exclusion criteria included: a) currently receiving an activated vitamin D analog or history of recent (< 3 months) use; b) currently receiving nutritional vitamin D (cholecalciferol or ergocalciferol) in dosages greater than 2000 IU/day; c) receiving erythropoiesis stimulating agents; d) receiving intravenous iron therapy or oral iron therapy started within 3 months prior to recruitment; e) severe anemia defined as a hemoglobin < 8.0 g/dl for males and < 7.0 g/dl for females; f) iron deficiency anemia defined as serum ferritin < 100 ng/ml and transferrin saturation < 20%; g) currently pregnant or breast feeding; h) screening serum calcium concentration > 10.0 mg/dl or serum phosphorus concentration > 4.5 mg/dl; i) acute kidney injury or rapidly declining GFR; j) receiving any form of renal replacement therapy including hemodialysis, peritoneal dialysis, or renal transplant; k) focus of active inflammation such as acute attack of gout or rheumatoid arthritis or active infection determined clinically.

**Fig. 1:**
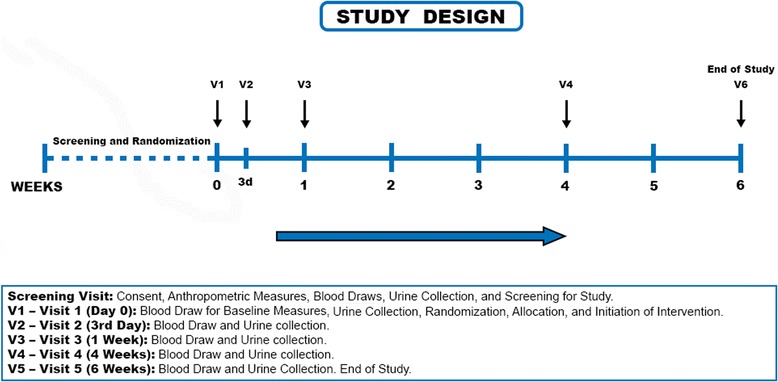
Overview of the study design

All study visits occurred on the Clinical Research Unit (CRU) of the Center for Clinical and Translational Science at UAB. Participants were randomized 1:1 to receive either oral calcitriol (0.5 mcg per day) or a matching placebo (one capsule daily) for a period of 6 weeks. Blinding was achieved by over-encapsulating calcitriol capsules to match placebo capsules. Blood parameters were obtained at baseline and at regular pre-specified time intervals to assess the impact of calcitriol vs. placebo on hepcidin concentrations, ferritin, iron, total iron binding capacity, calcium, phosphorus, intact parathyroid hormone (PTH) and hemoglobin (Fig. 1). The study was approved by the UAB Institutional Review Board for Human Use and all participants provided written informed consent. This study complied with Clinical Trial registration requirements (NCT1988116).

#### Outcomes of interest

The primary outcome of interest was the change in serum hepcidin concentrations in participants randomized to calcitriol as compared to those randomized to placebo. Quantitative measurements of bioactive 25-hepcidin were obtained in sera using a competitive enzyme-linked immunosorbent assay (ELISA, Intrinsic LifeSciences, La Jolla, CA) [12]. This assay uses anti-human hepcidin polyclonal antibody and biotinylated synthetic human hepcidin as a tracer and was developed in collaboration with the laboratory of Dr. Tomas Ganz. Assays are performed using proprietary polyclonal antibodies bound to 96-well immunoassay plates. The hepcidin concentration of each serum sample is indirectly measured by the competition between the amount of biotinylated hepcidin tracer present in each well and the amount of hepcidin present in the test sample. Following competition, the amount of tracer captured by the antibody in each well is detected by the addition of streptavidin-HRP. The colorimetric signal is detected at 450 nm and the optical density of each assay is used to quantify serum hepcidin using non-linear regression software based on a 12-point calibration curve employing synthetic hepcidin standards. The intra-assay coefficients of variation (CVs) of hepcidin are 11–19% in the lower range of hepcidin values (< 30 ng/ml) and 4–11% in the upper range of hepcidin values (> 30 ng/ml, the levels most commonly observed in CKD patients) and the corresponding inter-assay CVs were 12–40% in the lower range of hepcidin values and 2–10% in the higher range. Secondary outcomes of interest were change in serum ferritin, transferrin saturation, and hemoglobin concentrations in the calcitriol vs. placebo arms. Measurements of these latter variables were done using standard assays in the main UAB hospital laboratory.

### Study protocol - enrollment, randomization, and study visits (Fig. 1)

Potential participants were invited for a formal screening visit where written, informed consent was obtained by study staff after the study was explained in full. Potential participants who provided consent had height and weight measured and blood and urine samples were obtained for screening labs. Female subjects of child-bearing age (≤ 65 years) received a rapid urine beta-HCG test to rule out pregnancy. Eligibility to continue with the study was determined after the review of screening tests. Randomization and blinding of eligible participants was performed by the research pharmacy at UAB. Baseline anthropometric and laboratory variables were measured and participants began the study intervention after visit 1 (day 0). Urine samples (random) and fasting, morning blood samples were collected at pre-specified time intervals (visit 1 to visit 6) as outlined in Fig. 1. Data for the outcome variables as well as safety labs were collected at these time points. The study ended at 6 weeks after the final blood and urine samples were drawn.

A pre-specified safety event that required interruption of the study medication was the development of a serum total calcium concentration > 10 mg/dL at any of the follow-up visits. Participants whose serum total calcium concentrations increased above 10 mg/dL discontinued the study medicine and had serum total calcium concentrations re-checked until they normalized. Participants were allowed to re-enter the study and resume the study intervention if the serum calcium concentration normalized.

### Statistical analysis

To compare the baseline characteristics between the two groups, Student’s t-test or χ^2^ test were used for parametric and non-parametric variables respectively. Longitudinal changes in the primary outcome variables were examined using repeated measures linear mixed models. In these models, time and treatment group were the fixed variables and participants were modeled as random effect terms. We first tested for a time by treatment group interaction to determine whether the change in the outcome variables over time differed by treatment group assignment. If we detected a statistically significant interaction, we stratified the analysis by treatment group and tested the main effect of time in stratified models. If we did not detect a significant interaction, we removed the interaction term from the model and tested for the main effects of time and treatment groups. Hepcidin and ferritin concentrations were not normally distributed, so natural log-transformed values were used in all analyses. All analyses were conducted as intention-to-treat. A two-tailed *P* value < 0.05 was considered statistically significant. All analyses were conducted using SAS software version 9.4 (SAS Institute, Cary, NC).

### Sample size calculation

We powered the study to detect a significant difference in the change in serum hepcidin concentrations in participants randomized to calcitriol vs. placebo. In a prior study of 7 healthy individuals treated with ergocalciferol, serum hepcidin concentrations were noted to decrease by 34% within 72 h of receiving a single bolus dose. Given prior data from our group showing a mean hepcidin concentration of 120 ± 80 ng/ml in a population of stage 3–4 CKD patients [6], we estimated that we would need 17 participants in each treatment arm to observe a similar effect size (30% difference) in individuals randomized to the calcitriol vs. placebo arm, assuming a type I error of 0.05 and a power of 80%. In order to account for potential drop-out from the study, we recruited a total of 20 participants in each arm (40 participants overall).

## Results

**Fig. 2:**
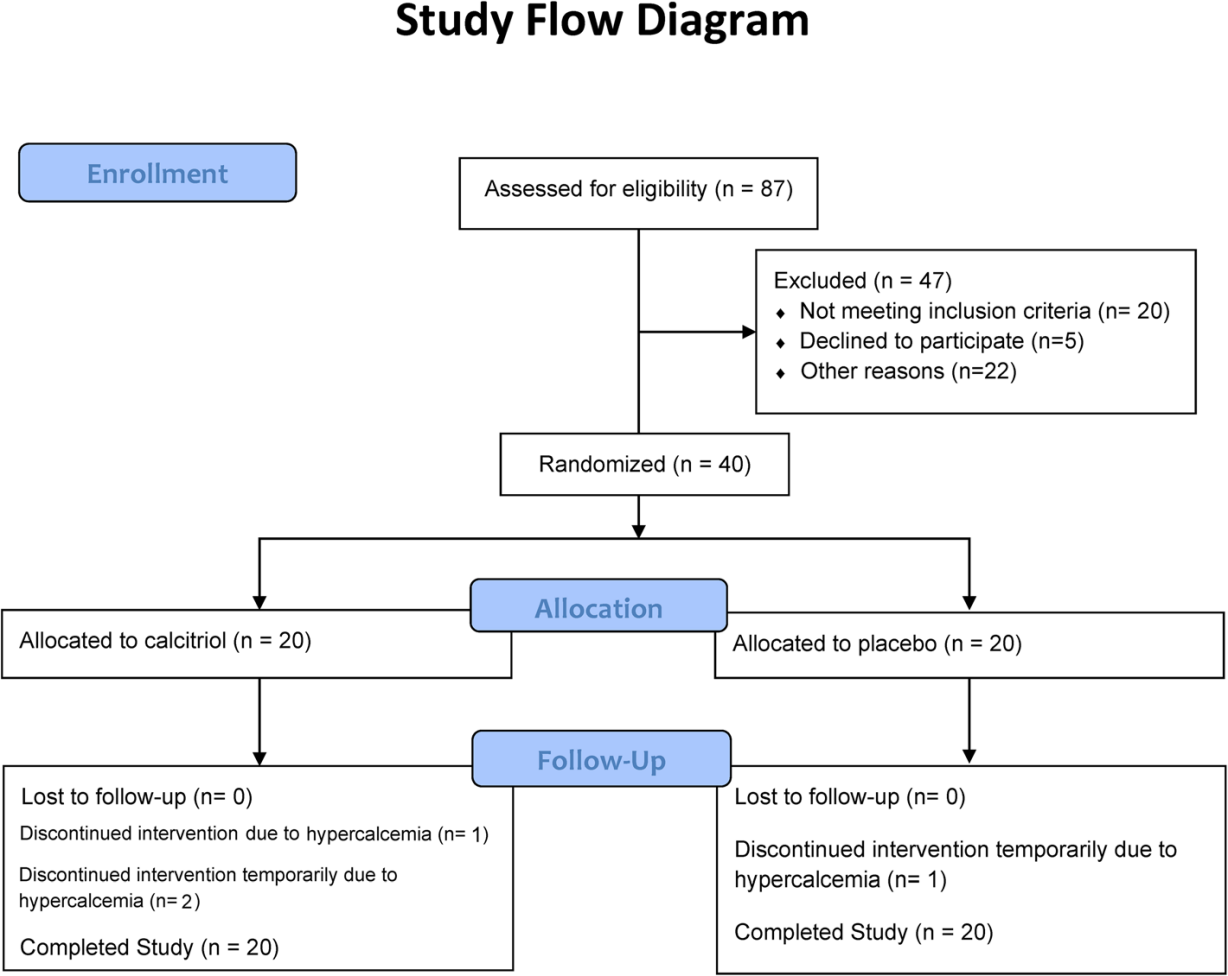
Study flow diagram depicting participant screening, enrollment, intervention allocation, and follow-up

Figure 2 depicts a study flow diagram summarizing the number of participants undergoing screening, enrollment, intervention allocation, and follow-up.

Baseline characteristics of the study sample by treatment group are shown in Table 1. A total of 20 participants were randomized to receive calcitriol and 20 were randomized to receive placebo. No participants were lost to follow-up. There were no significant differences in baseline characteristics between the two groups.

**Table 1:** Baseline characteristics of study participants by treatment group. Data are given as mean (SD), median [IQR] or frequencies

	Calcitriol	Placebo	P
N	20	20	
Age (years)	58.7 ± 9.4	62.4 ± 10.1	0.25
Male sex, N (%)	10 (50)	12 (60)	0.53
Black race, N (%)	12 (60)	15 (75)	0.31
BMI (kg/m^2^)	30.0 ± 6.2	33.4 ± 8.8	0.16
SBP (mmHg)	135.3 ± 26.6	130.1 ± 22.0	0.50
DBP (mmHg)	75.9 ± 12.3	71.8 ± 11.6	0.28
Laboratory variables			
Calcium (mg/dL)	9.1 ± 0.5	9.3 ± 0.3	0.14
Phosphorus (mg/dL)	3.87 ± 0.58	3.94 ± 0.65	0.22
PTH (pg/ml)	100.7 ± 73.6	108.4 ± 60.3	0.38
Hemoglobin (g/dL)	12.2 ± 1.9	13.3 ± 1.7	0.06
eGFR (mg/min)	35.8 ± 11.7	40.6 ± 10.8	0.19
UACR (mg/g)	254 [8, 2540]	39 [13, 188]	0.29
Hepcidin (ng/ml)	71.7 [46.5, 195.6]	76.1 [46.3, 124.1]	0.81
TSAT (%)	25.3 ± 12.5	23.2 ± 8.1	0.53
Ferritin (mg/dL)	193.5 [92.5, 374.5]	174.5 [66.0, 319.0]	0.34

*Abbreviations*: *BMI* Body mass index, *SBP* systolic blood pressure, *DBP* diastolic blood pressure, *eGFR* estimated glomerular filtration rate, *UACR* urine albumin to creatinine ratio, *TSAT* transferrin saturation

### Primary outcome - serum Hepcidin

**Fig. 3:**
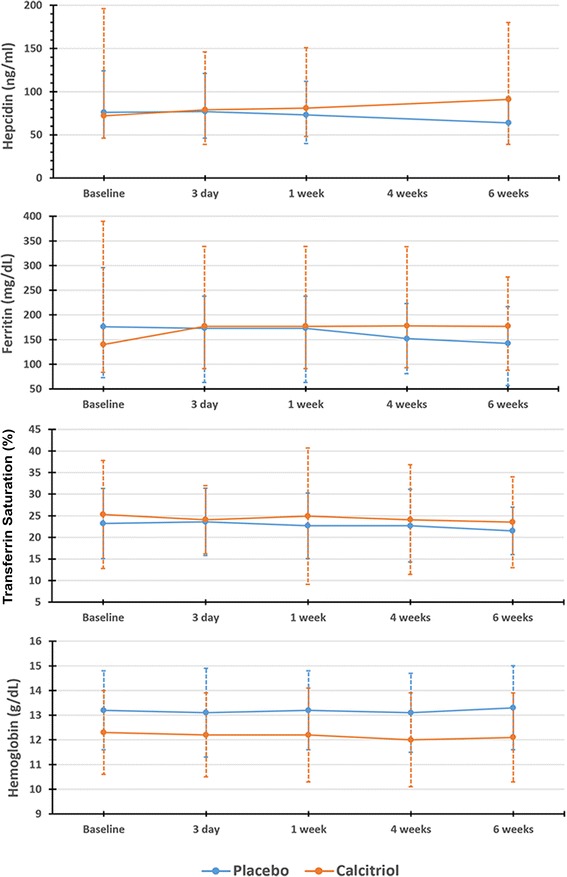
Change in serum hepcidin, median [IQR]; ferritin, median [IQR]; transferrin saturation, mean (±SD); hemoglobin, mean(±SD) over time by treatment group

Baseline median serum hepcidin concentrations were 71.7 [46.5, 195.6] and 76.1 [46.3, 124.1] in the calcitriol and placebo group respectively (*P* = 0.81). There was no statistically significant difference in the change in hepcidin concentrations over time by treatment group (*P*_*interaction*_ = 0.10) (Fig. 3). Additionally, there were no statistically significant changes in hepcidin concentrations over time (*P*_time_ = 0.19) or differences in hepcidin concentrations by treatment group (*P*_*group*_ = 0.46) during the six-week intervention.

### Secondary outcomes - hemoglobin, serum Ferritin and percent Transferrin saturation

There were no significant differences in the change in serum ferritin, TSAT or hemoglobin concentrations between treatment groups over time (*P*_*interaction*_ *>* 0.05 for all) (Fig. 3)*.* Similarly, there were no statistically significant differences in these outcome variables over time or by treatment group (*P* > 0.05 for all).

### Markers of mineral metabolism - serum intact PTH (iPTH), calcium and phosphorus

**Fig. 4:**
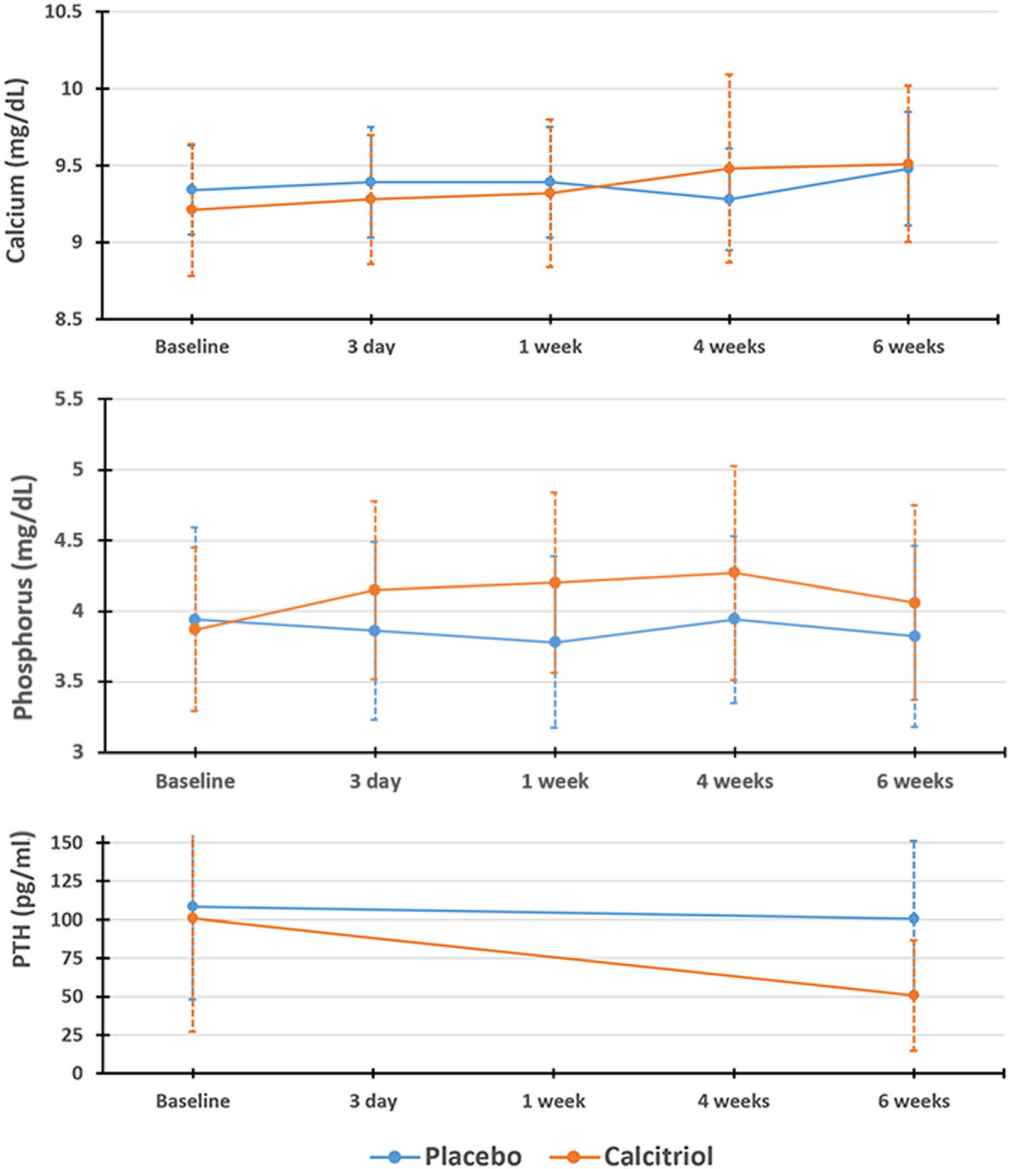
Change in mean(±SD) serum calcium, phosphorus, and PTH over time by treatment group

Baseline concentrations of serum calcium, phosphorus and iPTH were not different between the two groups. There were significant differences in the change in total calcium, phosphorus and iPTH concentrations over time between the two groups (*P*_*interaction*_ < 0.05 for all) (Fig. 4 and the Additional file 1: Table S1). In stratified models, serum total calcium significantly increased from baseline in the calcitriol group (*P* = 0.02), whereas no change in calcium was observed in the placebo group. Serum phosphorus concentrations significantly increased in the calcitriol arm (*P* = 0.03), but not in the placebo arm. Intact PTH, which was measured at baseline and at the end of 6 weeks of intervention, decreased in the calcitriol group (*P* = 0.002) while remaining unchanged in the placebo group.

### Safety measures

One participant in the placebo group and 2 participants in the calcitriol group had to discontinue the intervention temporarily due to hypercalcemia (Fig. 2). In addition, one participant in the calcitriol group had to stop the intervention one week prior to the end of the study due to hypercalcemia. When analyses were repeated excluding individuals who did not complete six weeks of therapy in full due to episodes of hypercalcemia (per protocol analysis), the results of the study did not change.

## Discussion

In this randomized, controlled trial, treatment with calcitriol for 6 weeks did not result in any significant changes in hepcidin, iron parameters or hemoglobin as compared to placebo in individuals with mild to moderate CKD.

Hepcidin modulates iron bio-availability by restricting its efflux from cells of the reticuloendothelial system and blocking iron absorption at the level of the enterocytes in the small intestine [3–5]. Elevated hepcidin concentrations are common in CKD patients [6–9], and are thought to play a key role in the development of anemia of CKD by reducing the amount of iron that is available for erythropoiesis [13, 14]. Experimental animal data show that reducing hepcidin improves anemia of chronic inflammatory diseases, suggesting that therapies that lower hepcidin may ameliorate anemia in CKD patients [15–17].

Vitamin D has recently been shown to modulate iron homeostasis via direct regulation of hepcidin synthesis. Bacchetta et al. showed that 1,25-dihydroxyvitamin D directly inhibits hepcidin expression by binding to a vitamin D response element in the gene encoding hepcidin (HAMP) [10]. In this same study, they showed that treatment with a single oral dose of ergocalciferol (100,000 IU) reduced hepcidin concentrations by 34% within 24 h of administration in seven healthy volunteers [10]. In line with these results, a study of patients with early stage CKD treated with high-dose ergocalciferol showed that the decline in hepcidin concentrations was directly proportional to the rise in 25-hydroxyvitamin D concentrations [18].

Based on these data, we hypothesized that treatment with vitamin D would reduce circulating hepcidin concentrations as compared to placebo in CKD patients. However, six weeks of calcitriol did not reduce serum hepcidin concentrations and did not have any impact on other markers of iron homeostasis as compared to the placebo in our study. There are a number of potential reasons for why we did not find any effect of calcitriol on the iron parameters in this study. We studied individuals with moderate to severe kidney disease, whereas prior studies enrolled healthy volunteers or individuals with very early CKD. In individuals with more advanced stages of CKD, hepcidin concentrations are elevated due to multiple factors (increased inflammation, reduced GFR, treatment with iron) that collectively could override any potential effect of vitamin D supplementation on inhibiting hepcidin expression. It is also possible that the nutritional forms of vitamin D that were used in prior studies are more efficacious in reducing hepcidin levels than the activated form (calcitriol) used in our study. This is because ergocalciferol provides the necessary substrate (25(OH)D) for cells that synthesize hepcidin (hepatocytes, monocytes) to produce 1,25(OH)_2_D for inhibition of hepcidin in an autocrine/paracrine fashion. Since individuals with CKD and even ESRD retain the capacity to convert 25(OH)D to 1,25(OH)_2_D in extra-renal sites, this may allow hepcidin-synthesizing cells the ability to down-regulate hepcidin synthesis according to physiologic need. In contrast, calcitriol replacement is largely non-physiological and mostly impacts circulating 1,25(OH)_2_D concentrations, which may not be most efficacious for regulating intracellular hepcidin synthesis. Next, it is possible that a higher dose of calcitriol is needed to effectively inhibit hepcidin synthesis in CKD patients. Importantly, however, participants treated with calcitriol had significant increases in serum calcium and phosphorus and a significant decrease in iPTH, indicating that the dose used in our study (0.5 mcg/day) was sufficient to effect physiological changes, at least with respect to mineral metabolism.

Other potential reasons for the lack of an effect of calcitriol treatment on hepcidin concentrations could include inadequate power to detect a more modest effect. While possible, the observation that serum hepcidin concentrations did not decrease at all during the period of intervention would suggest that even if calcitriol reduced hepcidin concentrations, the number needed to treat would be so high that it would have limited clinical efficacy. In addition, we did not exclude individuals with adequate iron status (TSAT ≥ 20 and Ferritin *≥* 100) in our study. This segment comprised 40% of our study population and may have diluted an effect of calcitriol on hepcidin and other markers of iron homeostasis since hepcidin may play a less prominent role in leading to anemia of CKD in these individuals as compared to those with iron-restricted anemia. In addition, we recruited all CKD patients irrespective of their vitamin D status. It is possible that the effects of vitamin D on hepcidin are more robust in individuals with vitamin D deficiency.

## Conclusions

In conclusion, 6 weeks of oral calcitriol did not decrease hepcidin concentrations in individuals with moderate kidney disease. Further studies should investigate whether nutritional forms of vitamin D are more effective in suppressing hepcidin secretion in CKD patients. If so, this may provide a novel strategy for reducing hepcidin and improving iron status in CKD patients.

## Additional file

Additional file 1: Table S1. Changes in study variables over time by treatment group. (DOCX 13 kb)
