# Supplementary material for: Effect of calcitriol on serum hepcidin in individuals with chronic kidney disease: a randomized controlled trial
Source: BMC Nephrol. 2018 Feb 9;19:35. doi: 10.1186/s12882-018-0823-7 (PMC5807766; doi:10.1186/s12882-018-0823-7)
Supplement: Supplementary file 1 — Changes in study variables over time by treatment group. (DOCX 13 kb) [file 12882_2018_823_MOESM1_ESM.docx]

|  |  | Baseline | 3 days | 1 week | 4 weeks | 6 weeks |
| --- | --- | --- | --- | --- | --- | --- |
| Hepcidin,  pg/ml, median [IQR] | Calcitriol | 72 [46,196] | 79 [39,146] | 81 [48,151] | -- | 91 [39,180] |
|  | Placebo | 76 [46,124] | 77 [46,121] | 73 [40,112] | -- | 64 [39,94] |
| Ferritin, | Calcitriol | 140 [84,390] | 177 [91,339] | 177 [91,339] | 178 [93,338] | 177 [88,277] |
| mg/dL, median [IQR] | Placebo | 176 [73,296] | 173 [63,238] | 173 [63,238] | 152 [81,223] | 142 [57,217] |
| Transferrin saturation, | Calcitriol | 25.3 ± 12.5 | 24.1 ± 7.9 | 24.9 ± 15.8 | 24.1 ± 12.7 | 23.5 ± 10.5 |
| %, mean + SD | Placebo | 23.2 ± 8.1 | 23.6 ± 7.8 | 22.7 ± 7.6 | 22.7 ± 8.4 | 21.5 ± 5.5 |
| Hemoglobin, | Calcitriol | 12.3 ± 1.7 | 12.2 ± 1.7 | 12.2 ± 1.9 | 12.0 ± 1.9 | 12.1 ± 1.8 |
| g/dL, mean + SD | Placebo | 13.2 ± 1.6 | 13.1 ± 1.8 | 13.2 ± 1.6 | 13.1 ± 1.6 | 13.3 ± 1.7 |
| Calcium, | Calcitriol | 9.21 ± 0.43 | 9.28 ± 0.42 | 9.32 ± 0.48 | 9.48 ± 0.61 | 9.51 ± 0.51^*^ |
| mg/dL, mean + SD | Placebo | 9.34 ± 0.29 | 9.39 ± 0.36 | 9.39 ± 0.36 | 9.28 ± 0.33 | 9.48 ± 0.37 |
| Phosphorus, | Calcitriol | 3.87 ± 0.58 | 4.15 ± 0.63 | 4.20 ± 0.64 | 4.27 ± 0.76 | 4.06 ± 0.69^*^ |
| mg/dL, mean + SD | Placebo | 3.94 ± 0.65 | 3.86 ± 0.63 | 3.78 ± 0.61 | 3.94 ± 0.59 | 3.82 ± 0.64 |
| PTH, | Calcitriol | 100.7 ± 73.6 |  |  |  | 50.7 ± 35.9^*^ |
| pg/ml, mean + SD | Placebo | 108.4 ± 60.3 |  |  |  | 100.4 ± 51.0 |

**Additional file 1: Table S1.** Changes in study variables over time by treatment group.
